# Intracerebroventricular injection of RFRP-3 delays puberty onset and stimulates growth hormone secretion in female rats

**DOI:** 10.1186/s12958-017-0254-5

**Published:** 2017-05-02

**Authors:** Xinghui Han, Yuanyuan He, Gulan Zeng, Yonghong Wang, Wen Sun, Junchao Liu, Yanyan Sun, Jian Yu

**Affiliations:** 1 Department of Integrative Medicine, Children’s Hospital of Fudan University, No.399, Wan Yuan Road, Min Hang District, Shanghai, China; 2 Department of Pediatrics, Xiamen Hospital of T.C.M, Xiamen, People’s Republic of China

**Keywords:** RFRP-3, Puberty onset, Kiss-1, GH

## Abstract

**Background:**

Puberty onset is a complex, organized biological process with multilevel regulation, and its physiopathological mechanisms are yet to be elucidated. RFRP-3, the mammalian ortholog to gonadotropin-inhibitory hormone, is implicated in inhibiting the synthesis and release of gonadotropin in mammals. However, it is unclear whether RFRP-3 participates in regulating pubertal development.

**Methods:**

This study investigated the functional significance and regulatory mechanism of hypothalamic RFRP-3 neuropeptide in the onset of puberty in young female rats. On postnatal day 22, we implanted cannulas into the lateral ventricles of female rat pups. From postnatal day 28 to postnatal day 36, the intracerebroventricular injection of RFRP-3, or vehicle, was conducted twice a day. To investigate whether puberty onset was affected, we examined the body weight, age of vaginal opening, serum hormone levels, uterus and ovary development, and hypothalamic Kiss-1 mRNA expression.

**Results:**

Intracerebroventricular injection of RFRP-3 significantly decreased the serum concentrations of luteinizing hormone and estradiol, delayed uterine maturation, and postponed the time of vaginal opening. This study suggests that RFRP-3 can delay the onset of puberty in young female rats; the expression of Kiss-1 mRNA is potently inhibited in the RFRP-3 group. Moreover, our data show that RFRP-3 elevates serum growth hormone levels.

**Conclusions:**

These data suggest that intracerebroventricular injection of RFRP-3 significantly delays the onset of puberty in female rats. Additionally, RFRP-3 may be associated with prepubertal rise in the secretion of growth hormone.

## Background

Puberty is the process of physical changes through which a child’s body matures into an adult body capable of sexual reproduction. The onset of puberty is a well-organized biological process controlled by the reproductive neuroendocrine systems [1, 2]. It is governed by high-frequency hypothalamic gonadotropin-releasing hormone (GnRH) pulsing [3–6]. The release of GnRH leads to the secretion of gonadotropins, luteinizing hormone (LH), and follicle-stimulating hormone (FSH), which target the gonads to trigger puberty. Previous studies reported that several upstream neuropeptides, such as kisspeptins [6–8], neuropeptide Y (NPY) [9], leptin [10], substance P (SP) [11], neurokinin B (NKB) [12–14], and RF-amide related peptide-3(RFRP-3) [15], were implicated in the control and modulation of GnRH secretion. However, the changes and interrelationships of these modulators before and during puberty remain poorly understood.

Kisspeptins, encoded by the hypothalamic Kiss-1 gene, are stimulators of the reproductive axis and play a critical role in the reproductive function [16, 17]. Kisspeptins potently elicit the release of gonadotropin, primarily through the stimulation of GnRH secretion. Numerous studies suggest that the kisspeptin/GPR54 system is the trigger for puberty onset [18–21].

RFRP-3, the mammalian ortholog of avian gonadotropin-inhibitory hormone (GnIH), is an inhibitor of the reproductive axis [22–25]. GnIH, which potently inhibits the release of pituitary gonadotropin, was first discovered in the avian species in 2000 [22]. RFRP-3 neuron fibers are found in close apposition to GnRH, kisspeptin, and growth hormone-releasing hormone (GHRH) neurons in the hypothalamus, suggesting a possible functional relationship among them [26–28]. Approximately 60–80% of GnRH neurons have RFRP-3 appositions in female prepubertal rats [29]. Additionally, GnRH and kisspeptin neurons can express the RFRP-3 receptor, GPR147 [28, 30]. This suggests that RFRP-3 may inhibit the reproductive axis by acting directly on kisspeptin neurons, GnRH neurons, or both. Furthermore, controversy remains over a possible direct effect of RFRP-3 at the level of the pituitary. RFRP-3 altered the levels of LH, it has been previously revealed. Several studies have demonstrated that the injection of RFRP-3 into the lateral ventricle, or intravenously suppresses LH secretion in adult rats, mice, hamsters, and sheep [25, 27, 30–33].

However, the above-mentioned studies were performed in adult mammals and only explored the instantaneous effect of a single injection of RFRP-3. There is little evidence showing that RFRP-3 plays a definite role in the timing of pubertal onset in young mammals. A study conducted in 2014 suggested that the presence of variants in the genes encoding human RFRP-3 and GPR147 is not associated with the occurrence of GnRH-dependent pubertal disorders [34]. The pubertal timing was not altered in GPR147 (NPFF1R) knockout (KO) mice [35]. These studies indicated that the RFRP-3/GPR147 pathway may play secondary, modulatory roles in the regulation of puberty onset. However, RFRP-3 pathways in the hypothalamus were associated with advanced puberty in female rats exposed to bisphenol A [29]. RFRP-3 injection significantly inhibited the release of LH, in an estradiol-dependent manner, in prepubertal female mice [36]. This suggested that hypothalamic RFRP-3 may be involved in the regulation of the reproductive axis in young animals.

Therefore, the modulatory effect of the RFRP-3 on the onset of puberty requires further investigation. In this study, we injected RFRP-3 into the lateral ventricles and compared the alteration in pubertal development in prepubertal female rats.

## Methods

### Animals

A total of 18 prepubertal female Sprague-Dawley rats (50–60 g), weaned on postnatal day (PND) 21, were purchased from the Shanghai SLAC Laboratory Animal Co., Ltd [license number: SCXK (Shanghai) 2012-0002]. The animals were housed in the animal center of the Children’s Hospital of Fudan University. The rats were provided with individual cages, standard rodent diet and water, and were maintained at an appropriate temperature (22 °C ± 2 °C) and humidity (55% ± 1.5%) and 12/12-h light/dark cycle. All procedures were approved by the Fudan University Animal Care and Use Committee in accordance with the National Institutes of Health Guide for Care and Use of Laboratory animals.

### Experimental design

Eighteen female rats were randomly divided into an RFRP-3 group, a Vehicle group, and a Normal group, with six rats per group. On PND 22, all the rats in the RFRP-3 and Vehicle groups received a cannula implantation into the lateral ventricle, while no treatment was performed on the rats in the Normal group. After the surgery, the rats were allowed to recuperate for 6 days to the cerebrovascular barrier to recover. From PND 22, the rats were weighed and the vaginal opening (VO) was observed every morning. From PND 28, the rats in the RFRP-3 group were injected with RFRP-3 at the dose of 0.5 μg/5 μl twice a day; the rats in the Vehicle group were injected with 5 μl physiologic saline twice a day; no treatments were performed on the rats in the Normal group. On PND 36, the rats were anesthetized with 5% chloral hydrate (0.8 ml/100 g body weight) and blood was drawn 30 min after the last intracerebroventricular(ICV) injection. Subsequently, the blood, hypothalami, uteri, and ovaries were immediately harvested.

### Cannula implantation into the lateral ventricle

On PND 22, a 3-mm cannula (Shenzhen RUIWODE Life Technology Co., Ltd, China) was inserted vertically into the lateral ventricle of the rats (AP = -0.8 mm, Lat = -1.2 mm, DV = -3.6 mm, relative to the bregma), and fixed, using dental cement, onto the surface of the skull. The correct location for the cannula implantation was determined histologically at the end of the procedure.

### ICV injection

RFRP-3 (also called NPVF; 048-33, Phoenix Pharmaceuticals, Inc., USA) was dissolved in sterile physiologic saline to a final concentration of 0.1 μg/μl. Polyethylene(PE) tubing was attached to a 3.6 mm inner pipe (Shenzhen RUIWODE Life Technology Co., Ltd) on one end and connected to a 10 μl microsyringe on the other end. The drug was slowly injected into the lateral ventricle and the inner pipe was gently extracted 10 min post-injection.

### Validation of the location of ICV injection

At the end of the procedure, the rats in the Vehicle and RFRP-3 groups were injected with 5 μl trypan blue into the lateral ventricles. After the hypothalamus was excised, the remaining brain tissue was fixed in 4% paraformaldehyde for 24 h. The brain tissue was sectioned at the thickness of 50 μm to assess whether trypan blue had been successfully injected into the lateral ventricle. Experimental data from each rat recorded whether the dye was successfully injected into the lateral ventricle.

### Uterine and ovarian histology

The uteri and ovaries were immediately excised and weighed to evaluate the organ coefficients, which were determined by the formula: [organ wet weight (g)/body weight (g)] × 10^-4^. The uteri and ovaries were fixed in 4% paraformaldehyde, and embedded in paraffin. The paraffin-embedded tissues were then sectioned at 4 μm and stained with hematoxylin-eosin (HE). The endometrial thickness was measured using a Leica microscope (four fields were selected to obtain the mean endometrial thickness).

### Serum hormone enzyme-linked immunosorbent assay

Blood samples were collected from the jugular veins and centrifuged at 1200 × g for 5 min; the serum was preserved in a -80 °C freezer. ELISA kits (eBioscience, Affymetrix, USA) were used to detect the concentrations of LH, FSH, estradiol (E2), and growth hormone (GH), according to the manufacturer’s instructions.

### Reverse Transcription Real-time Quantitative Polymerase Chain Reaction (RT-qPCR) for Kiss-1 and GnRH mRNA

The effects of ICV injection of RFRP-3 on the expression of Kiss-1 and GnRH mRNA in the hypothalami were detected by RT-qPCR. The whole hypothalamus was excised and snap-frozen in liquid nitrogen. Total hypothalamic RNA was extracted using a Direct-zol RNA MiniPrep kit (Zymo Research, USA) and reverse-transcribed into cDNA with an All-In-One RT Master Mix kit (ABM, Canada), according to the corresponding manufacturer’s protocols. All the reactions were performed in triplicate in a 20-μl total reaction volume. The following RT-qPCR amplification program was used: 95 °C, 3 min; then, 40 cycles of 95 °C, 5 s, and 60 °C, 30s(BIO-RAD Real-Time PCR Detection System). Glyceraldehyde-3-phosphate dehydrogenase (GAPDH) was used as the internal control, and mRNA levels were calculated using the 2^-△△Ct^ method. The primers were designed and synthesized by Shanghai Generay Biotech Co., Ltd, China (Table 1).

**Table 1:** Primers used in the study

Gene	Sequence (5′ to 3′)	Product size
GAPDH-F	ACTTTGGCATCGTGGAAGGG	128 bp
GAPDH-R	TGCAGGGATGATGTTCTGGG	
Kiss1-F	GGTATGCAGAGAGCAAGCCT	122 bp
Kiss1-R	GATCAGGCGACTGCGGG	
GnRH-F	CACTGGTCCTATGGGTTGCG	149 bp
GnRH-R	TCCCTAAGAGGTGAACGGGG	

### Statistical analysis

Generally, the data with a normal distribution and homogeneity of variance were compared using one-way ANOVA, and comparisons between groups were tested using Duncan’s multiple range tests. Otherwise, data were analyzed by the non-parametric Kruskal-Wallis test using SPSS software (Ver. 19.0; SPSS Inc.). The body weight, age at vaginal opening, wet weight of the uterus and ovary and their organ coefficients, serum hormone concentrations, and levels of Kiss-1 and GnRH mRNA were compared across groups by one-way ANOVA, with treatment as a factor. The results are presented as mean ± SEM. Statistical significance was assumed when *P* < 0.05.

## Results

### Effects of ICV injection of RFRP-3 on the body weight

**Fig. 1:**
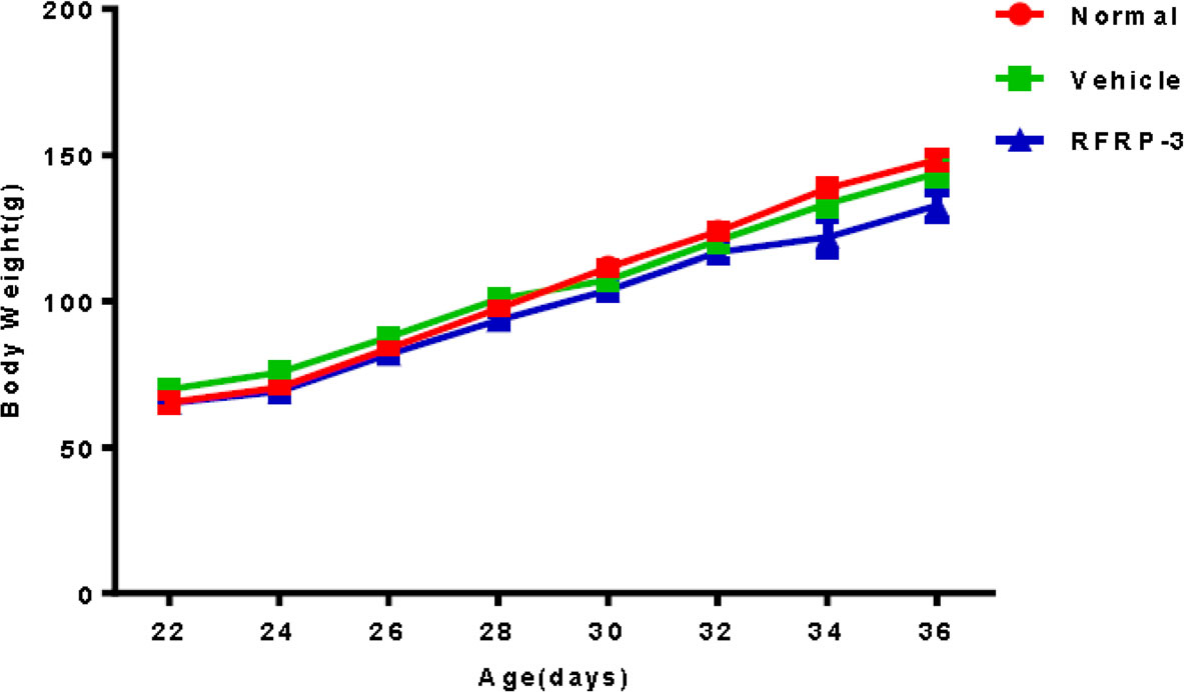
Effects of ICV injection of RFRP-3 on the body weight of female rats. No statistical significance was observed in the body weight among the three groups

As shown in Fig. 1, the body weight of rats in the different groups gradually increased with age. In all three groups, the body weight of the rats was not significantly different on PND22 (time of cannula implantation), PND28 (time of drug infusion), or PND36 (time of sampling) (Fig. 1, *P* > 0.05).

### Effects of ICV injection of RFRP-3 on the age at vaginal opening

**Fig. 2:**
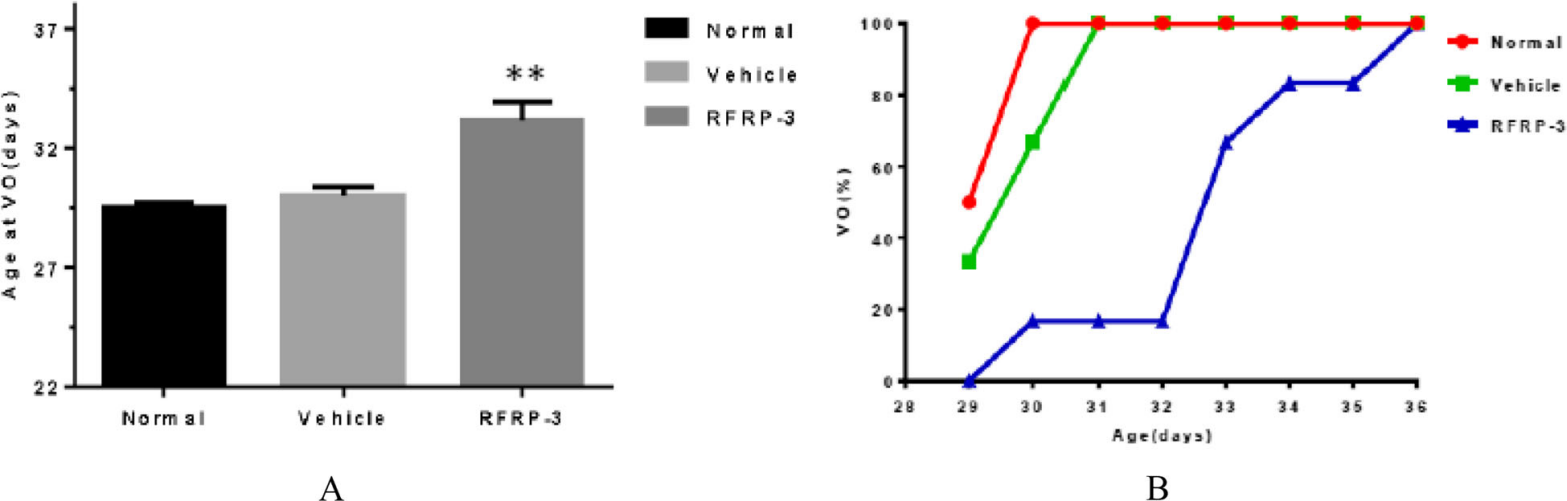
Effects of ICV injection of RFRP-3 on the age at vaginal opening. **a** The age at VO of the females in the different groups. Data represent means ± SEM. **P < 0.01 versus Vehicle. **b** The proportion of the females displaying VO at different postnatal ages for the different groups

The ICV injection of RFRP-3 exerted a significant effect on the age at vaginal opening (VO). All female rats exhibited vaginal opening before or at PND36. VO was significantly delayed in the RFRP-3 group (Fig. 2, *P* < 0.01), compared with that in the Vehicle group. The age at VO showed no obvious difference between the Normal and Vehicle groups (Fig. 2, *P* > 0.05).

### Effects of ICV injection of RFRP-3 on the wet weight of the uterus and ovary, and their organ coefficients

**Fig. 3:**
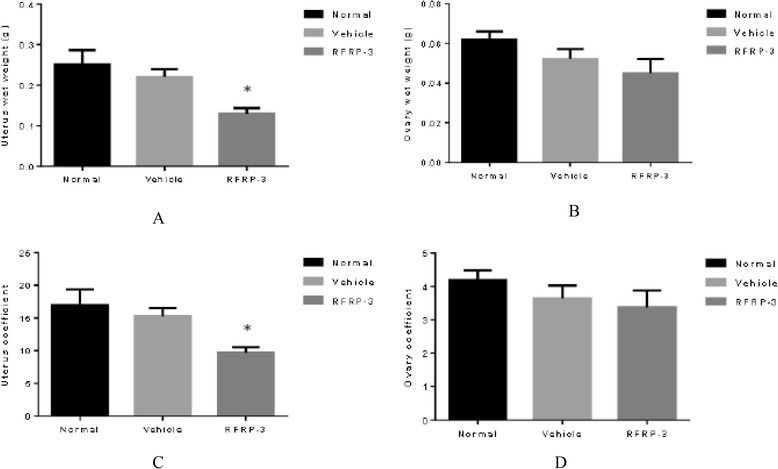
Effects of ICV injection of RFRP-3 on the wet weight of the uterus and ovary, and their organ coefficients. **a** Uterus wet weight. **b** Ovary wet weight. **c** Uterus coefficient. **d** Ovary coefficient. Data represent means ± SEM. *P < 0.05 versus Vehicle

Compared with the Vehicle group, ICV injection of RFRP-3 for 8 days resulted in a reduction of the uterus wet weight and coefficients (Fig. 3a and c, *P* < 0.05). There were no significant differences in the ovary wet weight and coefficients between the RFRP-3 and Vehicle groups (Fig. 3b and d, *P* > 0.05). Moreover, all observed indices showed no statistical differences between the Normal and Vehicle groups (Fig. 3, *P* > 0.05).

### Effects of ICV injection of RFRP-3 on morphological changes of the uterus and ovary

**Fig. 4:**
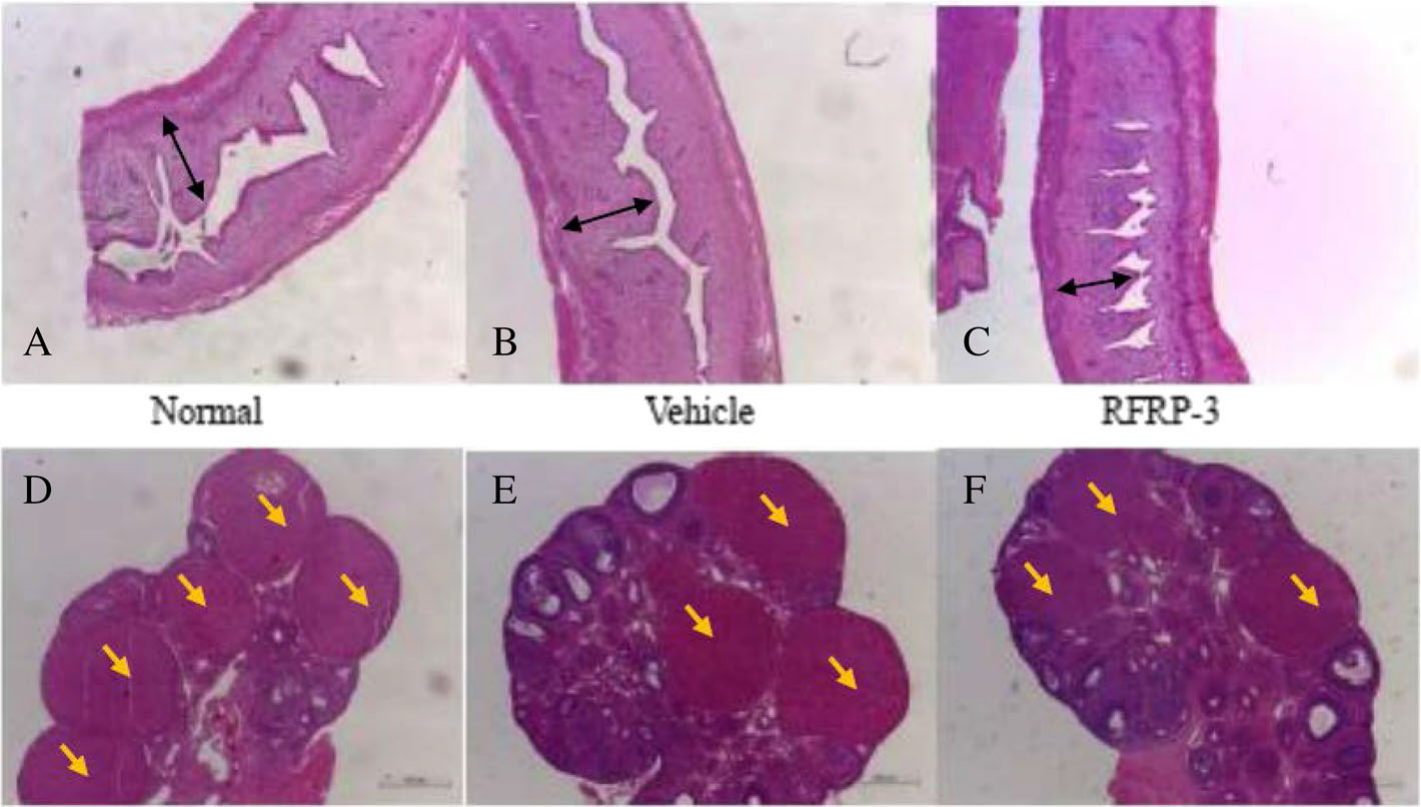
**a**, **b**, **c** Effects of ICV injection of RFRP-3 on uterine morphology. Compared with the Vehicle group, endometrial thickness (*black arrows*) of the RFRP-3 group was distinctly thinner. **d**, **e**, **f** Effects of ICV injection of RFRP-3 on ovarian morphology. Ovarian morphology was distinctly similar across groups. Numerous corpora lutea (*yellow arrows*) were observed in all three groups. Bar 500 μm

**Fig. 5:**
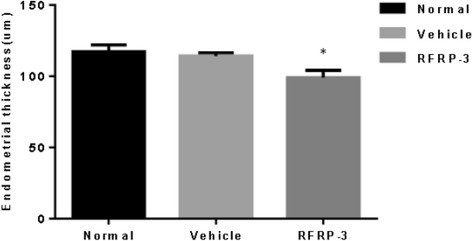
Effects of ICV injection of RFRP-3 on the endometrial thickness. Data represent means ± SEM. *P < 0.05 versus Vehicle

The results of HE staining of the uterus and ovary are shown in Fig. 4. A quantitative analysis of uterine morphology was also conducted (Fig. 5). The endometrial thickness of rats in the RFRP-3 group was thinner than that of the Vehicle group (Fig. 5, *P* < 0.05). There were no obvious differences in the endometrial thickness between the Normal and Vehicle groups (Fig. 5, *P* > 0.05). These data suggest that ICV RFRP-3 delayed uterine development. By contrast, ovary development was not affected by the injection of RFRP-3. The rats in the three groups presented consistent degrees of ovary development with abundant corpora lutea, and several antral and cystic follicles (Fig. 4d, e, and f).

### Effects of ICV injection of RFRP-3 on serum hormone levels

**Fig. 6:**
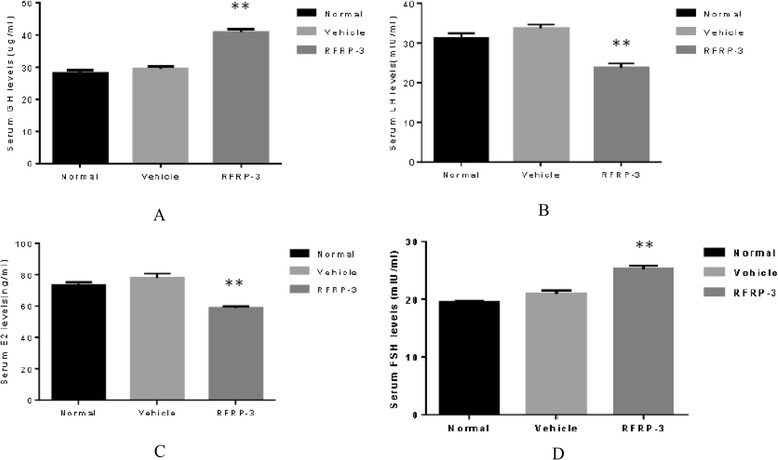
Effects of ICV injection of RFRP-3 on serum hormone levels. **a** Serum GH levels. **b** Serum LH levels. **c** Serum E2 levels. **d** Serum FSH levels. Data represent means ± SEM. **P < 0.01 versus Vehicle

ICV injection of RFRP-3 significantly decreased the serum levels of LH and E2 compared with those of the Vehicle group (Fig. 6b and c, *P* < 0.01). Serum levels of FSH were significantly elevated in the RFRP-3 group compared with those of the Vehicle group (Fig. 6d, *P* < 0.01). Additionally, ICV RFRP-3 also significantly elevated the serum levels of GH (Fig. 6a, *P* < 0.01). There were no observed differences in the levels of GH, FSH, LH, and E2 between the Normal and Vehicle groups (Fig. 6, *P* > 0.05).

### Effects of ICV injection of RFRP-3 on the expression of hypothalamic Kiss-1 and GnRH

**Fig. 7:**
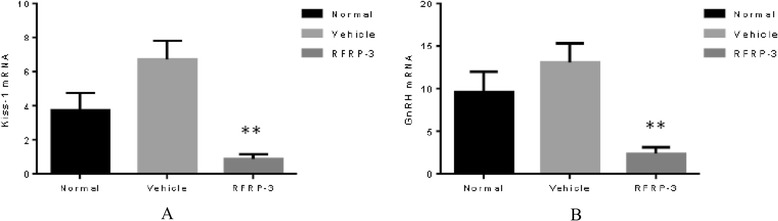
Effects of ICV injection of RFRP-3 on the expression of hypothalamic Kiss-1 and GnRH. **a** Kiss-1 mRNA level. **b** GnRH mRNA level. Data represent means ± SEM. **P < 0.01 versus Vehicle

To examine whether ICV injection of RFRP-3 can affect hypothalamic Kiss-1 and GnRH expression in prepubertal female rats, we used RT-qPCR to measure the abundance of the mRNA of the two genes. Figure 7 shows that ICV RFRP-3 clearly affects the mRNA expression of these genes. The mRNA levels of Kiss-1 and GnRH in the RFRP-3 group were significantly reduced compared with those of the Vehicle group (*P* < 0.01). There were no differences in the expression of Kiss-1 and GnRH between the Normal and Vehicle groups (*P* > 0.05).

## Discussion

This study assessed whether hypothalamic RFRP-3 neuropeptide had an inhibitory role in regulating the onset of puberty. We injected RFRP-3 into the lateral ventricles of prepubertal female rats twice a day for 8 days (from PND 28 to PND 36). To evaluate pubertal development, we observed the vaginal opening time, uterus and ovary development, and serum levels of sexual hormone. To explore the mechanism of action of RFRP-3, we detected the expression of hypothalamic Kiss-1 and GnRH mRNA. The status of the vaginal opening is a universally acknowledged external marker of female puberty onset in rodents. Our data show that ICV injection of RFRP-3 significantly delay the timing of puberty onset, characterized by delayed vaginal opening time and uterine development; it also reduce the serum levels of LH and E2. Recently, several findings suggested that endogenous RFRP-3 signaling may not be necessary for the timing of puberty [34, 35]. Pubertal analyses of null mice evidenced that GPR147 KO males, but not females, displayed constitutively elevated LH levels before and during puberty, whereas pubertal progression was not apparently altered by the congenital lack of GPR147 in either sex [35]. In accordance with this suggestion, a human genetics study revealed that the presence of variants in the genes encoding RFRP-3 and GPR147 is not associated with the occurrence of GnRH-pubertal disorders [34]. However, a 2015 study, by Semaan and Kauffman, showed a decrease in RFRP-3 expression in the hypothalamic dorsomedial nucleus during the prepubertal stage in mice [37]. This suggests that this decrease may disinhibit GnRH secretion and that RFRP-3 may be a possible inhibitory control during the onset of puberty. Consistent with that study, our results suggest that RFRP-3 delays the timing of puberty when administered exogenously, indicating that this peptide may play a significant role in sexual maturation and development.

Johnson et al. showed that ICV injection of RFRP-3 for 2 weeks reduced the levels of LH and wet weight of testes, but had no effect on puberty onset, in 35 day-old male rats [38]. The discrepancy in the results could be attributed to gender differences and/or to the RFRP-3 doses. Firstly, female rats were used in our study while male rats were used in the Johnson study. This raises questions on the possible sex-dependent differences in the role of RFRP-3 in the regulation of puberty onset. However, due to differences in experimental design, direct comparisons cannot be interpreted with confidence. Secondly, the dose of RFRP-3 was 1ug/day in our study and 15ug/day (15 times, 15 nmol/day, molecular weight: 990.15) in the Johnson study. In the past decade, numerous studies about RFRP-3 have been conducted in a variety of species with different developmental stages, different routes of administration, different doses, various treatment duration and also different molecules [either RFRP-3 (3-8) or entire molecules] [24, 27, 39, 40]. Several studies have demonstrated that injection of RFRP-3 decreases serum LH levels in a dose-dependent manner within a certain range [27, 39, 40]. However, higher doses can be ineffective on LH concentrations [24, 39]. Similarly, while the inhibitory action of RFRP-3 was observed at low RFRP-3 concentrations on GnRH stimulated LH secretion from pituitary cells, high concentrations had no effect [40]. So the relationship between the dose of RFRP-3 and inhibitory action on LH is more complicated than we anticipated. The use of different doses may be one of the reasons for the inconsistent results on with respect to the timing of puberty onset observed in Johnson’s study and the present results. Therefore, it is necessary to further investigate the relationship between different doses of RFRP-3 and the timing of puberty onset.

The various mechanisms, by which RFRP-3 may regulate the pubertal development, are not fully known. Previous findings suggested that RFRP-3 may directly or indirectly affect pituitary gonadotrophins and hypothalamic GnRH to regulate the reproductive axis. The Kiss-1 gene has been shown to be critical for the onset of puberty [19]. To determine whether Kiss-1 is a target for the central action of RFRP-3 in female rats, we examined Kiss-1 and GnRH mRNA expression in the hypothalamus. Accordingly, our data revealed that Kiss-1 and GnRH mRNA expression were significantly decreased in the RFRP-3 group. This raises a possibility that ICV RFRP-3 delays the timing of puberty onset in female rats mainly via a decrease in Kiss-1 expression, which is supported by several previous studies. RFRP-3 has been functionally shown to inhibit the electrical firing of some arcuate nucleus (ARC) kisspeptin neurons [41]. Rizwan et al. revealed that RFRP-3 can act at the rostral periventricular nucleus (PeN) kisspeptin and GnRH neurons to modulate reproduction, suggesting that RFRP-3 may directly regulate this kisspeptin population [28]. However, Poling’s study showed that RFRP-3 may directly modulate hypothalamic Kiss-1 neurons in mice but just a small proportion in the ARC [42]. They thought that the Kiss-1 and RFRP-3 likely act independently on the GnRH-pituitary axis and only have notable communication with each other at the level of RFRP-3 signaling to the ARC kisspeptin cells. Therefore, whether Kiss-1 is a major action site of RFRP-3 in the regulation of pubertal development is still controversial. Additionally, in our study, we excised the whole hypothalamus and measured whole hypothalamic Kiss-1 mRNA using RT-qPCR. It is preferable to separately measure the two primary kisspeptin populations of hypothalamic AVPV/PeN as well as ARC. Because of the limitation of our approach and the sophisticated molecular mechanisms of RFRP-3 regulatory actions, the various mechanisms involved in RFRP-3 mediated delay of pubertal timing are not fully known and require further investigation.

In our study, ICV RFRP-3 led to reduced secretion of LH and E2, but elevated FSH levels. This may be because RFRP-3 has no effect on FSH secretion; a single injection of RFRP-3 into lateral ventricular, or repeated injections for 14 days, affected LH levels, but had no effect on FSH levels, in adult male rats [27, 38]. This may be attributed to the time point of sample collection, which may not be optimal for the stimulation of FSH in rats. Another possible explanation for the decreased LH and increased FSH in the RFRP-3 group is a decreased GnRH pulse generator. The GnRH pulse generator is an important factor in regulating differential gonadotropin synthesis and secretion [43]. During the process of pubertal maturation, the responsiveness of LH and FSH to GnRH changes [43]. Before puberty, the predominant response is one of FSH secretion, while after puberty LH responses exceed those of FSH [43]. A decreased GnRH pulse generator in the group stimulated with RFRP-3 favors FSH secretion. Additionally, FSH levels remain constant during the onset of puberty (from PND15 to PND32) and decrease from PND32 to PND40 in normal Sprague-Dawley female rats [44]. These data may explain why FSH concentrations were higher in the RFRP-3 group than in the Vehicle group. These are also possible reasons for the delayed puberty onset and low uterine maturity, but normal ovarian maturity, in the RFRP-3 group; the uterus weight and morphology, not the ovary, are significantly changed during pubertal development in normal female rats. Studies show that the coefficient of the uterus, not the ovary, is significantly elevated in normal female rats between PND25 and PND40 [44]. On PND32 and PND40, the corpora lutea in rats are formed, and there is no significant difference in the number, indicating that no morphological changes occur during this period [44]. Thus, it is possible that RFRP-3 affects the time of puberty onset and the maturation of the uterus and ovaries, with a greater effect on the uterus. Additionally, puberty, a dynamic process of maturation of a young into a sexually mature adult, is controlled by a sophisticated network of regulatory signals. Our data suggest that RFRP-3 delays, but does not prevent the onset of puberty; therefore, it has limited effects on pubertal development. RFRP-3 may delay ovarian development, but not affect its final maturation.

Previous studies have shown that GHRH neurons are found in close apposition to the RFRP-3 neuron fibers in the hypothalamus, suggesting a possible functional relationship between them [27]. Of note, we found that RFRP-3 significantly promoted GH secretion. The serum GH level was significantly elevated 30 min after the last administration of RFRP-3 into the lateral ventricle, which was consistent with the data from Johnson in adult and 35 day-old male rats [38]. These results suggest that RFRP-3 may be an important regulator of growth during pubertal development in female rats. Gonadotropin releasing hormone analogue (GnRHa) has been used to treat precocious puberty; however, it alters the levels of GH, and then slows down the growth velocity [45–47]. GH and GnRHa combination therapy has been used to improve final height, but this therapy is costly [48]. In our study, RFRP-3 not only delayed puberty onset but also promoted the secretion of GH in female rats, which may provide a novel method for the treatment of precocious puberty.

## Conclusions

Our study is the first to reveal that ICV injection of RFRP-3 of prepubertal female rats can delay the timing of puberty onset. These results challenge the current notion of RFRP-3 exerting secondary, modulatory effects on mammalian puberty. This may not be as universal as previously assumed, as RFRP-3 appears to play a significant role in pubertal development. Additionally, RFRP-3 inhibited the central expression of hypothalamic Kiss-1 mRNA, suggesting that Kiss-1 may be one of the action sites in RFRP-3-delayed puberty onset. Moreover, in line with previous studies, we found that injection of RFRP-3 elevated the serum levels of GH, indicating that RFRP-3 can modulate the secretion of GH. These results suggest that RFRP-3 may be an important regulator for individual growth during pubertal development. Nevertheless, further research is needed to better characterize the specific mechanisms through which RFRP-3 delays the onset of puberty and alters the secretion of GH.

## Abbreviations

APVP: Anteroventral periventricular nucleus
ARC: Arcuate nucleus
E2: Estradiol
FSH: Follicle-Stimulating Hormone
GAPDH: Glyceraldehyde-3-phosphate dehydrogenase
GH: Growth hormone
GHRH: Growth hormone-releasing hormone
GnIH: Gonadotropin-inhibitory hormone
GnRH: Gonadotropin-releasing hormone
GnRHa: Gonadotropin releasing hormone analogue.
GPR54: G protein-coupled receptor 54
HE: Hematoxylin-eosin
ICV: Intracerebroventricular
KO: Knockout
LH: Luteinizing Hormone
NKB: Neurokinin B
NPY: Neuropeptide Y
PE: Polyethylene
PeN: Rostral periventricular nucleus
PND: Postnatal day
RFRP-3: RFamide-related peptide-3
RT-qPCR: Real-time Quantitative Polymerase Chain Reaction
SP: Substance P
VO: Vaginal opening
